# Studying Microbial Communities through Co-Occurrence Network Analyses during Processes of Waste Treatment and in Organically Amended Soils: A Review

**DOI:** 10.3390/microorganisms9061165

**Published:** 2021-05-28

**Authors:** José A. Siles, Mercedes García-Sánchez, María Gómez-Brandón

**Affiliations:** 1 Department of Plant and Microbial Biology, University of California at Berkeley, Berkeley, CA 94720, USA; josesimartos@gmail.com; 2 Department of Soil and Water Conservation and Waste Management, CEBAS-CSIC, 30100 Murcia, Spain; 3 Eco & Sols, CEDEX 2, 34060 Montpellier, France; garcia.sanchez.mercedes@gmail.com; 4 Grupo de Ecoloxía Animal (GEA), Universidad de Vigo, 36310 Vigo, Spain

**Keywords:** co-occurrence networks, composting, anaerobic digestion, digestate, soil organic amendments, soil microbial communities, sustainable agriculture

## Abstract

Organic wastes have the potential to be used as soil organic amendments after undergoing a process of stabilization such as composting or as a resource of renewable energy by anaerobic digestion (AD). Both composting and AD are well-known, eco-friendly approaches to eliminate and recycle massive amounts of wastes. Likewise, the application of compost amendments and digestate (the by-product resulting from AD) has been proposed as an effective way of improving soil fertility. The study of microbial communities involved in these waste treatment processes, as well as in organically amended soils, is key in promoting waste resource efficiency and deciphering the features that characterize microbial communities under improved soil fertility conditions. To move beyond the classical analyses of metataxonomic data, the application of co-occurrence network approaches has shown to be useful to gain insights into the interactions among the members of a microbial community, to identify its keystone members and modelling the environmental factors that drive microbial network patterns. Here, we provide an overview of essential concepts for the interpretation and construction of co-occurrence networks and review the features of microbial co-occurrence networks during the processes of composting and AD and following the application of the respective end products (compost and digestate) into soil.

## 1. Introduction

### 1.1. Strategies for the Recycling of Organic Wastes

The activity of the ever-increasing human population generates millions of tons of solid organic wastes (that is, any material that is biodegradable and comes from living organisms) [1]. The inappropriate handling of these wastes is leading to soil, water and air contamination [2]. Therefore, finding strategies to reuse and treat organic wastes is of utmost need [3]. Organic wastes have the potential to be used as a resource for renewable energy production and as a source of soil nutrients, which not only helps to reduce our dependence on inorganic fertilizers, but also represents an ecologically-sound and economically attractive alternative to landfill disposal and incineration [4]. This is especially relevant in the current attempts to reach a fully circular economy [5]. 

Broadly, organic wastes can be classified into the following categories: (i) animal manures: solid, semisolid and liquid by-products generated by animals grown to produce meat, milk, eggs and other agricultural products; (ii) plant residues: crop wastes including stalks and stubble (stems), leaves, green manures and seed pods; (iii) waste from manufacturing processes: by-products coming from different industrial activities such as exhausted seeds, hoof and horn meal, animal feathers and fur, wastes from sugar extraction, distillery waste and biosolids from paper mills; and (iv) biosolids: treated wastewater residues from municipal wastewater treatment plants [6,7]. Some of these have the potential to be used as soil organic amendments and/or as a raw material to generate energy by anaerobic digestion (AD) [2]. However, their direct application into soil may lead to harmful effects such as environmental contamination, increasing ammonia volatilization, decreasing soil oxygen concentration, producing phytotoxic compounds and immobilizing soil mineral N [8,9,10]. Additionally, certain wastes have undesirable characteristics such as odor, pathogens, toxins and other contaminants (pharmaceutical compounds, endocrine disruptors, heavy metals, etc.) [11,12]. Therefore, most wastes need to be stabilized through biological processes such as composting in order to transform them into safer end products with benefits for both agriculture and the environment [1]. Alternatively, AD is an effective procedure to recover energy from organic wastes by converting the input materials into a renewable energy resource, namely biogas [2]. This technology is especially interesting since not only does it produce biogas, but also a stabilized product known as digestate that has the potential to be revalorized as an organic amendment [13]. The application of compost and digestate into soil has been proposed as an effective way of improving soil quality and fertility, while at the same time, protecting the environment because their use could be part of a strategy to eliminate and recycle massive amounts of waste [8].

### 1.2. Composting and Anaerobic Digestion

Composting involves the aerobic humification of biomass by microorganisms under controlled conditions [14]. Under optimal conditions, three phases can be identified during the process. A first mesophilic phase, which takes place under 40 °C and where there is an explosive growth of mesophilic microbes at the expense of easily degradable compounds such as sugars, carbohydrates and amino acids. A second thermophilic phase occurs above 40 °C and compounds such as hemicellulose, cellulose, fats and lignin are degraded by thermophilic microorganisms in periods lasting from days to months. This phase allows the sanitization of the waste by the removal of pathogenic microorganisms. The third and last phase is a maturing stage, which is characterized by the decrease in the temperature to the mesophilic range, a slower decomposition rate of the remaining recalcitrant substrates and the enrichment of the compost in humic substances (i.e., humification) [1,11]. Proteobacteria, Firmicutes, Bacteroidetes and Actinobacteria, among Bacteria, and Ascomycota, among Fungi, have shown to be the predominant groups during composting processes, with their relative abundances changing depending on the starting materials and the type of composting procedure [15,16,17]. The duration of the composting process is highly variable, ranging from two months (for easily degradable wastes) up to several years (for highly recalcitrant materials). Among the main factors controlling the speed of the process are moisture content, C:N ratio of the material, temperature, oxygen (aeration), pH and size of the compost pile [18,19].

AD involves the anaerobic processing of raw wastes using a microbial consortium composed of hydrolytic, acidogenic and acetogenic bacteria as well as methanogenic archaea that work sequentially during the process, generating biogas (~70% CH_4_ and 30% CO_2_) and a by-product called digestate [20,21]. The first phase of the process is a hydrolysis where carbohydrates, proteins and lipids are converted into glucose, amino acids and fatty acids. Acidogenesis is the second phase and consists of the transformation of the hydrolyzed chemical compounds into acetate, propionate, butyrate, hydrogen, CO_2_ and ammonia. At the final phase, called methanogenesis, hydrogen, acetate and CO_2_ are converted into CH_4_ [1,22]. The duration of a typical two-stage digestion varies between 15 and 40 days [23]. At the end of the process, a by-product called digestate is generated. This by-product contains significant amounts of residual organic C and nutrients for plants, which confers its potential to be used as an organic amendment [24].

### 1.3. Benefits of Using Soil Organic Amendments

Intensive farming is characterized, among other practices, by the indiscriminate use of inorganic fertilization [25]. The continuous and over application of agrochemicals involves environmentally concerning side effects such as poor nutrient-use efficiency, enhanced greenhouse gas emissions, groundwater eutrophication and soil organic matter (SOM) loss [26,27]. Reduced SOM contents usually lead to a worsening in soil fertility and structure as well as in soil biodiversity, which results in a deterioration of soil quality and land degradation [28]. Sustainable agriculture, as an alternative to agricultural intensification, advocates for the application of organic amendments such as compost and digestate to maintain and improve soil structure and fertility [29]. On the other hand, agricultural soils have been recognized as an important hotspot for climate change mitigation owing to their great potential to act as C (carbon) sinks [30]. An appropriate approach to increase contents of sequestered C in soils relies on the application of organic amendments since a part of the added C is stabilized in the long term through physical, chemical and biochemical mechanisms [31].

Organic amendments improve SOM levels and have both direct and indirect beneficial effects on soil properties [7]. They have shown to improve the physical, chemical and biological fertility of soil [8,32]. Increases in SOM contents lead to enhanced soil aggregate stability and other properties such as soil porosity, water infiltration, water holding capacity and percolation, thus improving physical fertility [8,33]. Although each amendment has specific cation exchange capacity characteristics, it has been demonstrated that soil cation exchange capacity increases after the application of amendments [34,35]. This is vital for retaining essential nutrient cations and making them available to plants [36]. Likewise, organic amendments are responsible for enhancing other soil properties related to chemical fertility such as pH, electrical conductivity and the availability of essential nutrients, such as N, P and K, for plant growth [35,37]. It has also been reported that the application of organic amendments improves the status of microbial communities in terms of abundance, diversity and activity (biological fertility) [32,38]. At the plant level, the continuous release of nutrients from the added soil inputs can sustain the microbial biomass and activity for longer periods of time, resulting in higher plant nutrient availability in the long term [39]. In fact, the global meta-study of Luo et al. [32] demonstrated that crop yield is 27% higher upon application of soil organic amendment (farmyard manure, compost, green manure, solid waste and straw) than mineral fertilization. The use of soil amendments also has the potential to enhance the biocontrol of soil-borne diseases and soil dwelling pests [40]. In general, the beneficial aspects of organic amendments depend on the specific properties of the amendments, the application doses and the stabilization degree of the added materials [41,42].

### 1.4. Importance of the Study of Microbial Communities in Processes of Waste Transformation and in Organically Amended Soils

As previously mentioned, the role of archaea, bacteria and fungi is critical during composting and AD since they mediate the conversion of organic matter through a variety of chemical reactions [1]. Microbial communities in organic wastes are diverse and dynamic and are often metabolically closely linked to each other [16,43]. Therefore, studying microbial communities and their interactions is crucial to attain the successful treatment of the organic fraction of wastes. Knowledge on the microbial communities inhabiting the different types of compost and digestate is also useful to predict their potential impact on soil fertility [44]. For example, mature compost has shown to be rich in free-living N_2_ fixers and phosphate solubilizers, plant growth promoting microbes and enzyme-producing bacteria [19]. On the other hand, the implementation of sustainable agricultural practices such as the addition of organic amendments into soil needs to assess the impact of such practices, not only on soil physicochemical properties, but also on soil microbial communities since they play a pivotal role in the functioning of the entire ecosystem [45]. In this context, the characterization of soil microbial communities in terms of abundance, functionality and diversity in organically amended soils is crucial. Their study has shown that the use of soil organic amendments generally increases bacterial and eukaryotic diversity while synthetic fertilization reduces it [46].

Nowadays, high-throughput sequencing has produced a leap forward in the understanding of microbial communities, providing insightful data on microbial occurrences and dynamics under different scenarios. The most commonly used approach is metataxonomics, that is, the sequencing of marker gene amplicons (e.g., 16S rRNA (ribosomal ribonucleic acid), ITS (internal transcribed spacer), 18S rRNA, etc.) [47]. The data obtained can be used to: (i) describe the composition of microbial communities, (ii) calculate alpha-diversity measures (richness, Shannon index) and (iii) apply multivariate statistical techniques such as clustering and ordination (β-diversity) to compare processes of composting, AD or soil management practices [48,49]. It is now clear that microbial-mediated functions are more than just collective traits of different individuals, but the interactions among them [50]. Therefore, to move beyond the classical analyses of metataxonomic data and to dig deeper into the microbial interactions within a community, co-occurrence network approaches have become of increasing interest within microbial ecology studies [48]. The modelling of a co-occurrence network for a community can be useful to learn about specific interactions among its members, to identify key members, predict robustness to external alterations and modelling the environmental factors that drive microbial network patterns, among others [51]. In light of the useful information that networks analyses can provide on microbial communities’ behavior, microbial ecologists are starting to apply them to their metabarcoding studies. Therefore, the main objective of the present work was to give an overview about the potential of network analyses as a proxy/tool to evaluate the co-occurrence (i.e., interactions) patterns among microbial communities in processes of composting and AD and in soils amended with the resulting products (i.e., compost and digestate).

## 2. Characterizing Microbial Communities through Co-Occurrence Networks

In a given microbial community, individual members interact with one another to form a complex network of positive, negative and neutral interactions [52]. By interacting among each other, microorganisms drive biogeochemical cycles, influence plant growth, mineralize SOM or degrade contaminants, among other processes [50]. In recent years, network analyses have been used to visualize co-occurrence among the members in communities of microorganisms [52]. Networks—that is, a collection of nodes (community members) establishing interactions (represented as links) among them as a system—are useful to find details on community assembly rules reflecting ecological processes (mutualism, competition, predation, etc.), habitat filtering as well as mathematical coupling among different populations that regulate system functions in space or time [53,54]. If environmental and physicochemical conditions are included in the network analyses, the results reveal which conditions the microbial co-occurring assemblages of organisms prefer or avoid [55]. The implementation of networks analyses on microbial ecology has been possible thanks to the massive amount of data produced by the high-throughput sequencing technologies.

Several tools, such as differential equation-based, Bayesian and relevance/co-expression network approaches, have been developed to conduct network analyses in genomic biology [51]. Among them, the correlation-based relevance network method is the most commonly used in microbial ecology due to its simple calculation procedure and its robustness to noise. Spearman or Pearson correlation coefficients are recommended to be used to identify the co-occurrence links within a community [56]. However, some studies use arbitrary correlation thresholds to construct the networks [57,58], which leads to the creation of subjective rather than objective networks. To solve this problem, tools based on random matrix theory have been developed in order to automatically define an objective correlation threshold [51,59]. Most of the current studies analyzing microbial communities in processes of waste treatment or in organically amended soils through network analyses are based on metabarcoding data; i.e., pairwise correlations of the relative abundance of OTUs (operational taxonomic units) or ASVs (amplicon sequencing variants) across different samples are calculated [51]. The networks can be intra-kingdom, when only the interactions among the members of the same microbial kingdom of the community are considered (e.g., only bacterial 16S rRNA metabarcoding data are used for the analysis); or inter-kingdom, when the interaction among the members of different kingdoms of the microbial community are considered (e.g., when 16S rRNA and ITS metabarcoding data are merged or 18S rRNA metabarcoding data are used). In general, inter-kingdom co-occurrence networks have shown to be more informative [60]. Despite not being very common, networks revealing co-occurrence patterns of genes [61] (i.e., using metagenomics, functional gene arrays or metatranscriptomics data) and proteins (metaproteomics) [62] are also interesting tools to investigate microbial communities from a functionality perspective.

**Figure 1:**
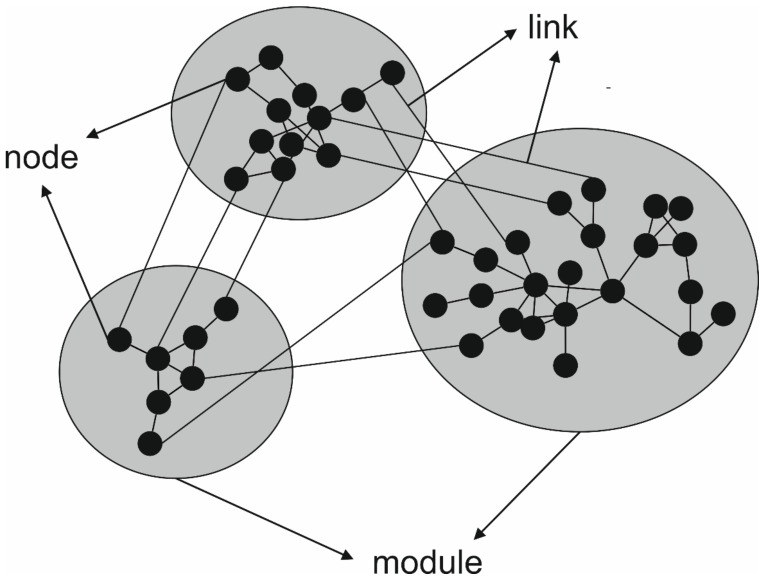
Schematic representation of a taxonomic co-occurrence network. Each circle represents a node (i.e., microbial OTU/ASV). A line connecting two nodes represents a positive or negative interaction between them (link). Nodes grouped into gray zones represents modules (i.e., clusters of densely interconnected nodes).

When a network is constructed, significant positive and negative interactions among all the nodes in the network are determined and graphically represented (Figure 1), and different topological properties are calculated to characterize the network as a whole and each of the nodes. Some of the more common topological properties used to describe networks and nodes have been summarized in Table 1. The number of nodes in the network indicates the number of connected OTUs/ASVs. This number is different from richness in most of the communities since the connection of some nodes will be null, meaning that their abundance is independent from that of other nodes. The proportion of positive and negative links provides information about the density of the interactions and the general behavior of the network [63]. The complexity of a network (i.e., connectivity and density) is a key topological property and it refers to the total number of nodes and links in the network. A greater network complexity has been related to microbial communities with a more intense activity and a higher resilience to perturbation [63]. Likewise, microbial networks of greater complexity are characteristic of the least disturbed ecosystems. For example, Banerjee et al. [64] demonstrated that agricultural intensification leads to a reduction in soil microbial network complexity. Connectedness is the capacity of a network to link two nodes taken at random by at least one link. From an ecological point of view, connectedness is an indicator of community cohesion [63]. Modularity measures the degree to which the network is organized into clearly delimited modules, which are clusters of densely interconnected nodes (Figure 1). It is another useful index to investigate the resistance of communities to disturbance [58]. Changes in the relative abundance of microbial populations with strong module memberships are probably driven by the same underlying factors. Thus, it is reasonable to hypothesize that the microbial populations with strong module memberships are physically and/or functionally associated in a microbial community [65]. This means that modularity helps control disturbances by compartmentalizing social–ecological systems [53].

The most useful indices to characterize nodes are (i) connectivity, which is the number of links of a node to other nodes; (ii) path length, which is the shortest path between two nodes; (iii) the clustering coefficient, which describes how well a node is connected with its neighbors; and (iv) betweenness centrality, which indicates the number of the shortest paths that pass through the node among the shortest paths existing between every pair of nodes [56]. Betweenness centrality has been shown to be useful to identify keystone nodes. According to the definition of Banerjee et al. [66], microbial keystone taxa are highly connected taxa that individually or in a guild exert a considerable influence on the microbiome structure and functioning irrespective of their abundance across space and time. These taxa have a unique and crucial role in microbial communities, and their removal can cause a dramatic shift in microbiome structure and functioning. However, according to Röttjers and Faust [67], networks do not offer any empirical demonstration that an OTU or ASV identified as a keystone by network co-occurrence is actually a keystone taxa. Therefore, in networks analyses, it is important to distinguish between keystone OTUs/ASVs (those identified through computational inference) and keystone taxa (those with explicit experimental evidence for their keystone role in the community) [66]. Keystone OTUs/ASVs exhibit the topological features of the shortest edge paths, high closeness centrality, and high betweenness centrality in co-occurrence networks. Likewise, keystone OTUs/ASVs can also be identified by module memberships, as explained by Deng et al. [51].

A typical co-occurrence network analysis in a microbiome study usually comprises a graphical representation of the networks along with the calculation of the aforementioned network topological indices and the identification of keystone OTUs/ASVs for each of the networks being constructed. Furthermore, direct comparisons of networks and their topological features are made in order to gain insights into, for instance, the effect of a specific treatment on microbial network patterns. Factors driving differences between samples or experimental treatments can be elucidated by applying Mantel and partial Mantel tests correlating the network topology and the changes in physicochemical or environmental conditions [51].

## 3. Features of Microbial Co-Occurrence Networks in Processes of Composting and Anaerobic Digestion

### 3.1. Composting

Exploring the interactions among microbial taxa coexisting in composting matrixes has not received enough attention yet [49]. Putting the accent on this matter is of utmost importance because depending on the degree of these interactions and the species involved, the dynamics of the composting process may vary with further consequences on the quality of the end product and its potential usefulness as an organic amendment. Recent studies have shown a predominance of positive interactions within co-occurrence microbial networks in composting systems [49,60,68,69], suggesting that potentially commensalistic or mutualistic interactions take place throughout the process. These positive interactions might play an important role during composting, since, for instance, the degradation of recalcitrant material requires the synergistic action of specialized groups like cellulose- and/or lignin-degrading microorganisms [60].

On the contrary, the occurrence of negative relationships in a network may be attributed to parasitism, antagonism, predation or competition [70]. Zhu et al. [60] found that the proportion of negative correlations for bacteria–fungi links (inter-kingdom competition) was higher than those between bacteria–bacteria and fungi–fungi (intra-kingdom competition) during the co-composting of reed straw with nitrogen-rich substrates. This points towards a possible competition for resources among the bacterial and fungal members of the community, which can result in a selective competitive pressure on each other. Biological inhibition caused by the production of antimicrobial substances may be another factor leading to the occurrence of negative correlations over the course of the composting process [71]. The co-occurrence patterns of bacterial and fungal communities can also be altered by the presence of additives in the composting system. Lei et al. [72] found that adding superphosphate and phosphogypsum resulted in a more complex and well-connected bacterial network, while reducing the fungal network size, during the composting of swine manure. Similar results were reported by Bello et al. [49] by adding biochar during cattle manure–maize straw composting. By using additives, it might yield conditions where microbes need more energy to survive promoting the competition for food resources through the various composting stages [72].

The complexity and density of microbial co-occurrence networks may depend on the initial compost feedstocks. It has been shown that nutrient-rich input materials may result in more complex co-occurrence networks with greater connectivity [60]. This can be indicative of a more stable and resilient microbial community [64], but further case studies are needed to corroborate and generalize this observation in composting matrixes. The removal of the keystone OTUs from the network and/or changes in the number of associations that these OTUs share amongst them can greatly influence the compost microbiome composition and the dynamics of the composting process [73]. Keystone populations show a greater capacity for nutrient exchange and a stronger resistance when environmental conditions become more limiting [68]. The initial feedstock and the operational conditions used for composting will largely influence which keystone OTUs will comprise the co-occurrence network [73]. Previous studies have reported keystone microbes from the phyla Firmicutes and Actinobacteria through different phases of the composting process [68,69,73]. While the phylum Actinobacteria is associated with oligotrophic environments, the phylum Firmicutes is mainly considered a fast-growing copiotroph group. Both oligotrophic and copiotrophic bacterial groups mainly differ in their growth strategies under nutrient-rich conditions and their efficiency in metabolizing carbon substrates, i.e., labile and recalcitrant compounds for copiotrophs and oligotrophs, respectively [74]. Specifically, the genera *Bacillus* and *Ureibacillus* from the phylum Firmicutes appeared as a keystone during the thermophilic phase of dairy manure composting [73]. These keystone OTUs positively correlated with certain physicochemical parameters such as temperature, NH_4_^+^ concentration and pH. Actinobacteria from the genera *Nonomuraea*, *Actinomadura*, *Mycobacterium* and *Sphaerisporangium* were more characteristic of the composting maturation phase [73]. These genera were positively associated with the germination index (GI), C/N ratio and NO_3_^−^ concentration, all of them used as proxies of compost maturity [75]. Alpha- and Gammaproteobacteria from the genera *Rhizobiales* and *Pseudomonas*, respectively, were also identified as keystone OTUs when maize straw was used as the raw material for co-composting with green soybean hulls (*Pseudomonas*; [69]) and seaweed (*Rhizobiales*; [68]). Pseudomonas OTUs were positively associated with GI [69], which is in line with the fact that certain *Pseudomonas* strains are known to confer plant-disease suppression and have the potential of nitrogen fixation favouring plant growth [76,77]. All in all, it reinforces the potential of co-occurrence networks as useful tools to understand how microbe–microbe interactions change throughout the composting process in response to environmental parameters, and how these changes might influence the quality of the end product by identifying the keystone OTUs that have a large influence in the community.

The evaluation of fungal co-occurrence network patterns during composting remains underexplored compared with bacteria [72]. Ascomycota and Basidiomycota are considered the dominant phyla in lignocellulose degradation and their high abundances are beneficial for organic waste decomposition during composting [60,69,72,78,79]. Based on the aforementioned studies, *Chaetomium* sp., *Penicillium* sp., *Trichoderma* sp., *Trichosporon* sp., *Arthrographis* sp. and *Aspergillus* sp. constitute the core fungal genera during composting. These fungi are in general efficient lignocellulose degraders and, like in the case of *Aspergillus* sp., some species can also promote compost maturation [80]. The fungal community succession during composting was shown to be mostly influenced by the temperature, pH, total N and/or organic matter content [69,72,81,82].

### 3.2. Anaerobic Digestion

The comprehensive understanding of the microbial communities in terms of interactions between network patterns and operational parameters, biodiversity and system functions is crucial to decipher the factors governing the stability and performance of the AD bioreactors [83]. Previous efforts have primarily focused on the roles of individual populations in process stability, especially on methanogens [84]. However, the study of co-occurrence networks established by microbial communities in AD reactors has permitted the acquisition of new knowledge about the successional pattern of microbial communities and their positive/negative interactions. For instance, by using RMT-based approaches, Wu et al. [85] showed that the microbial communities’ interactions in AD-bioreactors operating continuously for two years have a clear successional pattern, exhibiting increased modularity but decreasing the connectivity between populations over time. This study also revealed the microbial phylogenetic diversity as the most important factor associated with the network topology, indicative of induced niche differentiation over time [85]. The positive correlation between phylogenetically similar OTUs is usually due to niche overlap, as phylogenetically-related microorganisms are likely to behave similarly in niche adaptation [86]. Phylogeny could shape positive interactions, but not negatives ones, which suggests that the negative associations likely resulted from stochastic processes [87]. The observed increase of modularity over time reflected a great segregation within the microbial communities into finer niches and functional units, which in turn indicated a strong niche differentiation resulting in weaker interactions between microbial communities [70]. On the other hand, the positive correlation between phylogenetic diversity and network modularity suggested that network modularity is related to niche differentiation since it is essential in maintaining population diversity [88].

Ecological network analysis has also been used to better understand how biogas-producing microbiomes are constructed based on specific operational conditions such as the organic loading rate (a measure of the quantity of influent substrate entering the digester per unit of time), the hydraulic retention time (the average length of time that a soluble compound remains in an AD bioreactor), temperature (the mesophilic vs. thermophilic conditions) and the nature of the feedstocks [89]. For example, the organic loading rate is a critical operational parameter that must be controlled in order to avoid an upset in the bioreactors [90]. Usually, an organic loading rate shock produces an imbalance between hydrolysis/acidogenesis and the methanogenesis steps. As a consequence, higher amounts of volatile fatty acids are accumulated, decreasing the pH and leading to methanogenesis failure [91]. A recent study has found that the organic loading shock of a sewage sludge anaerobic reactor by adding glycerol waste resulted in the accumulation of volatile fatty acids after only 24 h [92]. The process led to the formation of 9 modules of co-occurring microorganisms with different behaviors during overloading, as revealed by a network analysis. Thus, the relative abundance of the *Veillonellaceae* family (glycerol degrading) and *Candidatus Cloacimonetes* (volatile fatty acids fermenters), was found in detriment of the syntrophic bacteria. Consequently, the methanogenesis failed 72 h after organic overloading, when the pH reached values lower than 6. Overall, the network analysis provided useful information about a succession of functionally redundant microorganisms, most likely because of niche specialization during organic overloading.

Xu et al. [83] revealed that changing organic loading rates were responsible for finer microbial network modules in comparison with different hydraulic retention times, suggesting a certain subdivision of functional components. Under the different conditions tested, a high proportion of nodes taxonomically classified as Firmicutes were positively correlated with biogas production, which points out the key role of this group of bacteria in the regulation of AD. The network analysis also showed that the core microbiome was similar under the different organic loading rates and hydraulic retention times studied, which would indicate that this overlap microbiome of generalist microorganisms is responsible for maintaining the ecological stability of bioreactors [83].

Temperature also plays a crucial role in the microbial interactions that affect the stability and performance of AD [93]. Based on a microbial network analysis, Lin et al. [94] indicated that there were more positive interactions between hydrogenotrophic methanogens (i.e., *Methanobrevibacter* and *Methanobacterium*), as well as substrate-hydrolyzing and hydrogen-producing bacteria (such as *Clostridium*, *Tepidimicrobium* and *Syntrophomonas*) at elevated temperatures (50 °C), resulting in an enhancement of CH_4_ production. The variations of microbial interactions in terms of network modularity and deterministic processes based on topological features corresponded well with the variations of CH_4_ productions, but not with the variation of temperatures. A common successional pattern of microbial interactions was observed at different temperatures, which showed that both the deterministic processes and the network modularity increased over time during the digestion process, improving the stability and efficiency of the AD-bioreactor.

The nature of the feedstocks has also been pointed out to play a key role in determining not only the network topological properties in AD bioreactors, but also the taxonomic affiliation of the keystone OTUs [95]. Vendruscolo et al. [96] compared the microbial networks inhabiting an upflow anaerobic sludge blanket reactor fed with bovine manure and a continuous stirred tank reactor fed with swine manure. Keystone OTUs in the first bioreactor were taxonomically identified as *Candidatus Cloacomonas*, *Methanospirillum* and *Methanosphaera*, while in the second one they belonged to the archaeal groups *Methanobrevibacter* and *Candidatus Methanoplasma*. This study also pointed out the presence of *Parcubacteria uncultured bacteria*, *Candidatus Cloacomonas* and *Candidatus Methanoplasma* as the keystone OTUs to maintain AD functioning, regardless of the type of manure.

## 4. Features of Microbial Co-Occurrence Networks in Compost- and Digestate-Amended Soils

Sustainable agriculture advocates for the use of environmentally-sound practices that enhance and/or maintain soil fertility [29]. The addition of compost and digestate into soil has been shown to not only improve the chemical and physical attributes of soil fertility but also the biological ones (i.e., soil living organisms) [97]. In this regard, it is well documented that amending soil with compost and digestate usually leads to increased microbial abundance, taxonomic diversity and enzymatic activity in an extent dependent on the properties of the added materials [8]. Altogether, it will potentially impact the direction and the intensity of the interactions that community members establish among them in concomitance with changes in the composition and functionality of microbial communities [98,99]. Therefore, microbial co-occurrence networks analyses seem to be a useful approach to conduct an integrative assessment of the suitability of compost and digestate as organic amendments in the context of soil biological fertility, as demonstrated by the increasing body of literature on this topic.

In this regard, the application of compost amendments into soil has been shown to increase network complexity for both bacteria and fungi when compared to inorganic fertilization and/or unamended treatments [100,101,102,103]. A similar trend in co-occurrence networks has been reported for other types of amendments like manure and straw [104]. Generally speaking, an increased network complexity might point towards the presence of a more interactive, stable and resilient soil microbial community to external pressures in organically amended soils [105]. Particular emphasis has been placed on microbial groups that carry out key functions in the agroecosystems, namely, those affecting nitrogen turnover rates. Yang et al. [100] observed that the complexity of both ammonia oxidizers and *nirS* containing denitrifiers networks gradually increased along with the compost rate. This is of particular importance for the productivity of plants given that nitrification and denitrification are among the most important soil N transformations.

In addition, amending soil with compost was shown to enhance the synergistic interactions within bacterial and fungal co-occurrence networks [99]. These latter authors found that fungal networks in a soybean agroecosystem harbored more positive links among saprotroph–saprotroph and saprotroph–symbiotroph when compost was applied into a moderate dose within a single growing season. It is likely that the external inputs of organic matter have resulted in more heterogeneous niches for soil microbes and thus have alleviated the competition for limited resources. In other words, a greater supply of nutrients following compost application might lead to more opportunities for different species to interact with each other. Nonetheless, under long-term fertilization regimes, Liu et al. [102] observed that using compost amendments did increase network complexity in the kiwifruit rhizosphere but promoted antagonisms and competition between microbial taxa, especially bacteria. Fewer correlations between the soil variables and the keystone microbes were also reported in the compost networks by Liu et al. [102], which is consistent with other studies [103]. This fact might suggest that keystone members are expected to be less affected by environmental perturbations in compost amended soils, which is in line with the increased network complexity in response to compost addition.

Nevertheless, research on the impact of digestate on soil microbial network patterns is still in its infancy. In a recent study from Tang et al. [106], they demonstrated that bacterial networks were more complex in soils amended with liquid digestate, as indicated by a higher number of nodes and links in the network, when compared to inorganically fertilized soils. This was explained by the authors in relation to the highly diverse type of nutrients provided by liquid digestate, which counterweigh the influence of niche partitioning in the microbial community assembly by allowing more stochastic processes to occur, while niche overlap introduces more relationships and interactions. Instead, fungal networks were found to be less complex than the bacterial ones [70,106]. Liquid digestate application probably led to a filtering effect on the fungal network and decreased its complexity, which might be caused by the competition between soil bacteria and fungi [107]. Moreover, its application into soil might have also favored more stable communities as revealed by the greater average degree, clustering coefficient and shorter average path distance in the respective bacterial and fungal networks [106]. This finding can be explained by the fact that a nutrient-rich environment can accelerate the growth of soil microorganisms and the underlying evolutionary process would generate more functional traits instead of promoting the community stability through functional diversification [108,109]. Keystone OTUs in the bacterial networks were taxonomically classified as Chloroflexi, while fungal keystone OTUs belonged to the genera *Ceratobasidium* and *Typhula*. Interestingly, Tang et al. [106] significantly correlated network modules with the different soil organic carbon (SOC) fractions and concluded that the application of liquid digestate stimulated the participation of soil bacteria and fungi in SOC mineralization and its chemical composition.

## 5. Concluding Remarks

Co-occurrence network analyses are starting to be applied with success to metataxonomic data in surveys studying the microbial communities driving processes such as compositing and anaerobic digestion and in organically amended soils. This approach represents a step forward in our understanding of the interactions among the members of a microbial community and the identification of its keystone members. Likewise, the existing works have shown that the study of network patterns is useful to decipher the environmental factors that control a specific process (e.g., composing, AD or enhanced soil fertility) by modulating the network properties of a microbial community. Most of the existing studies on networks consider only bacterial communities; thus, further studies also considering archaeal and fungal communities are needed. The application of microbial network approaches to metagenomic data can also provide a new perspective in the functional study of microbial communities. Co-occurrence networks thus have potential to become default analyses in microbial ecology studies.

## Figures and Tables

**Table 1:** Common topological indexes used to characterize networks and nodes. Adapted from Dai et al. [50] and Deng et al. [51].

Index	Meaning
**Overall network topological indexes**	
Connectivity	Connection strength between nodes, higher connectivity means a more complex network.
Geodesic distance/Path length	Shortest path between two nodes.
Density	How completely the network is populated with links. It is closely related to connectivity.
Connectedness	It is 0 for networks without links and is 1 for a connected graph. It is one of the most important measurements for summarizing hierarchical structures.
Modularity	Tendency of a network to contain sub-clusters of nodes.
Transitivity	Probability that two nodes are both directly and indirectly (using another node) connected.
Maximal degree	The maximal connection strength between nodes.
**Indexes for individual nodes**	
Connectivity	It is the connection strength between nodes and serves as an important measurement for summarizing hierarchical structures.
Edge paths	It shows paths between any two nodes in the network.
Mean degree	It counts the mean number of links per node in network.
Closeness centrality	It explains the average distance of one node to any other node.
Betweenness centrality	It reflects the number of times a node plays a role as a connector along the shortest path between two other nodes.
Clustering coefficient	It measures the extent of the connection between a node and its neighbor nodes in the network.
